# Hyperparameter optimization for cardiovascular disease data-driven prognostic system

**DOI:** 10.1186/s42492-023-00143-6

**Published:** 2023-08-01

**Authors:** Jayson Saputra, Cindy Lawrencya, Jecky Mitra Saini, Suharjito Suharjito

**Affiliations:** Industrial Engineering Department, BINUS Graduate Program - Master of Industrial Engineering, Bina Nusantara University, Jakarta 11480, Indonesia

**Keywords:** Cardiovascular disease, Data-driven analytics, Data mining, Hyperparameter optimization, Orange data mining software, Prognostic system, Unsupervised machine learning

## Abstract

Prediction and diagnosis of cardiovascular diseases (CVDs) based, among other things, on medical examinations and patient symptoms are the biggest challenges in medicine. About 17.9 million people die from CVDs annually, accounting for 31% of all deaths worldwide. With a timely prognosis and thorough consideration of the patient’s medical history and lifestyle, it is possible to predict CVDs and take preventive measures to eliminate or control this life-threatening disease. In this study, we used various patient datasets from a major hospital in the United States as prognostic factors for CVD. The data was obtained by monitoring a total of 918 patients whose criteria for adults were 28-77 years old. In this study, we present a data mining modeling approach to analyze the performance, classification accuracy and number of clusters on Cardiovascular Disease Prognostic datasets in unsupervised machine learning (ML) using the Orange data mining software. Various techniques are then used to classify the model parameters, such as k-nearest neighbors, support vector machine, random forest, artificial neural network (ANN), naïve bayes, logistic regression, stochastic gradient descent (SGD), and AdaBoost. To determine the number of clusters, various unsupervised ML clustering methods were used, such as k-means, hierarchical, and density-based spatial clustering of applications with noise clustering. The results showed that the best model performance analysis and classification accuracy were SGD and ANN, both of which had a high score of 0.900 on Cardiovascular Disease Prognostic datasets. Based on the results of most clustering methods, such as k-means and hierarchical clustering, Cardiovascular Disease Prognostic datasets can be divided into two clusters. The prognostic accuracy of CVD depends on the accuracy of the proposed model in determining the diagnostic model. The more accurate the model, the better it can predict which patients are at risk for CVD.

## Introduction

Diseases related to the circulatory system impact the blood vessels and coronary arteries, and are prevalent globally. In developed nations, they are the primary cause of mortality in grown-ups. It is crucial to diagnose heart ailments with precision and timeliness by taking into account a patient’s medical history and lifestyle. This approach enables accurate prognosis and the implementation of preventive measures to manage or eradicate these potentially fatal illnesses [1].

According to the 2013 Global Burden of Disease report by The Lancet, chronic illnesses pose the highest risk among all human ailments. Contributing factors comprise immoderate alcohol intake, hypertension, gender, and age. While these illnesses are widespread in affluent nations like the United States, where they account for 87% of fatalities, developing countries with lower and middle incomes require particular consideration due to the escalating incidence of chronic illnesses [2].

**Fig. 1:**
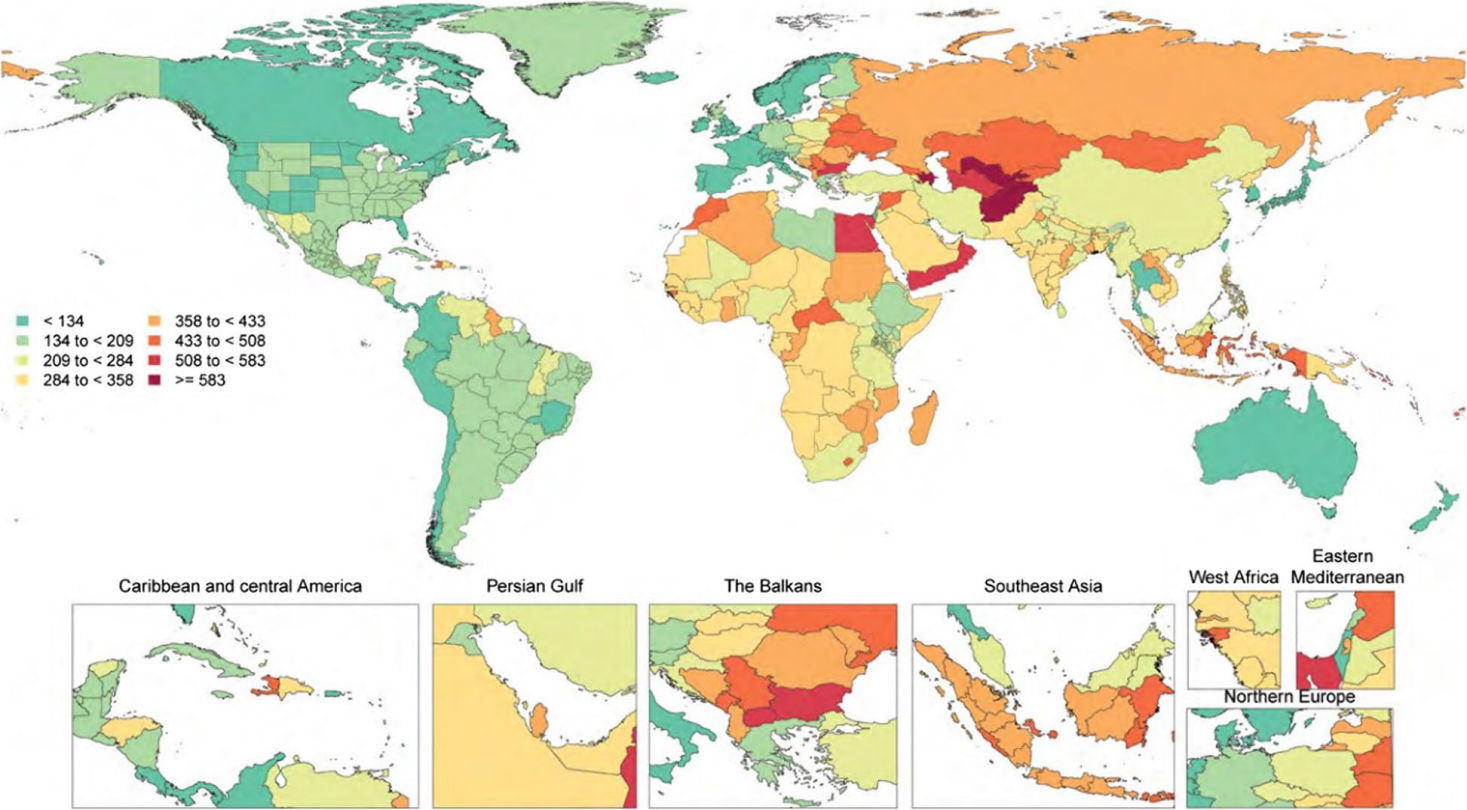
CVD mortality rates are expected to increase significantly by 2020 [3]

During the year 2020, the regions with the maximum age-adjusted rates of mortality caused by cardiovascular disease (CVD) were Eastern Europe, Central Asia, Oceania, North Africa, the Middle East, Central Europe, Sub-Saharan Africa, and South and Southeast Asia. Conversely, the regions with the minimum age-adjusted CVD mortality rates were high-income Asia-Pacific and North America, Latin America, Western Europe, and Australasia. Figure 1 indicates the age-standardized mortality rates per 100000 individuals affected by CVD across all countries [3].

**Fig. 2:**
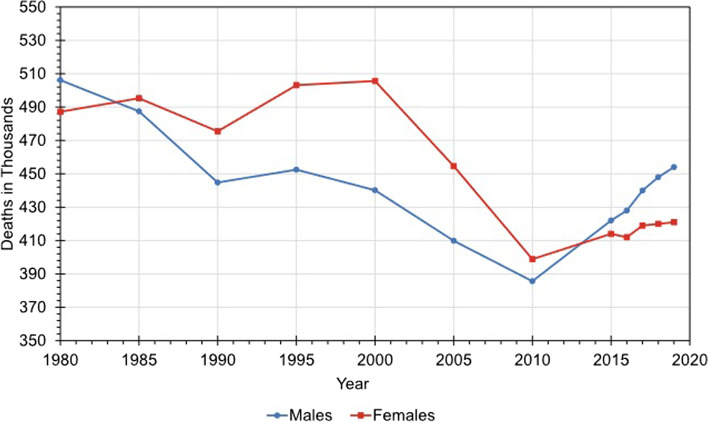
Trends in CVD mortality for men and women in United States from 1980 to 2019 [3]

The CVD fatality count in the United States declined from 1980 to 2010, but in recent times, it has escalated from 78454 in 2010 to 874613 in 2019. Figure 2 illustrates the patterns [3].

In 2019, coronary artery disease (41.3%) emerged as the primary reason for fatalities caused by CVD in the United States. Following this, stroke (17.2%), hypertension (11.7%), heart failure (HF, 9.9%), coronary heart disease (2.8%), and various other minor causes (17.3%) were observed [3].

CVDs make up 31% of the total fatalities globally, where 75% of the deaths occur in low- and middle-income nations. In wealthier nations, there is a higher occurrence and fatality rate of CVDs among individuals belonging to lower socioeconomic backgrounds [4]. Smoking, alcohol consumption, low fruit and vegetable intake, high salt intake, sedentary lifestyle, obesity, air pollution, genetic and metabolic factors, and other medical conditions are risk factors for CVD [5].

Forty percent of deaths in China are caused by CVD, a result of the aging population and a rise in stable metabolic risk factors. It’s crucial to lower the prevalence of CVD through primary prevention, allocate more medical resources for emergency and critical care, and offer rehabilitation and secondary prevention services to decrease the chances of relapse, re-hospitalization, and disability in CVD survivors. In China, millions of individuals are affected by hypertension, dyslipidemia, diabetes, and vascular diseases, including myocardial infarction and stroke, are frequently diagnosed [6].

CVD continues to be the primary reason for illness and death across the globe, even with regional management measures [7]. The Morbidity and Mortality Conference has evolved into a valuable resource for surgeons to scrutinize complications and introduce changes to avert recurrence. Such insights can effectively curtail ‘preventable’ adverse outcomes among both novice and seasoned surgeons [8].

The role of gender in health and disease is gaining importance, yet there is a dearth of systematic gender research in the field of medicine. Women are at a greater relative risk of suffering from CVD-related morbidity and mortality compared to men, primarily due to conventional factors such as obesity, hypercholesterolemia, hypertension, and diabetes, along with socio-economic and psychosocial factors, including depression. Additionally, depression amplifies the likelihood of CVD in women [9].

The coronavirus disease of 2019 (COVID-19) pandemic has altered the customary treatment for non-hospitalized individuals and those with sudden cardiac conditions, with the suspension of non-essential surgeries and a decrease in the effectiveness of current emergency medical services. In response to this crisis, novel methods like telehealth, online platforms, mobile apps, and artificial intelligence (AI) are being employed [10].

The pathophysiology of inflammation, blood clotting, and heart muscle damage linked with Severe Acute Respiratory Syndrome Coronavirus 2 can be evaluated by utilizing circulating biomarkers. Increased levels of cTn and NPs detected individuals with a higher probability of experiencing cardiovascular events while hospitalized, whereas increased levels of D-dimer detected individuals at risk of developing blood clotting issues [11].

People who have contracted COVID-19 are more prone to developing CVDs, such as disorders affecting the blood vessels in the brain, irregular heartbeats, heart diseases caused by reduced blood flow to the heart muscle, inflammation of the sac surrounding the heart, inflammation of the heart muscle, HF, and blood clots obstructing blood vessels. These hazards and difficulties are noticeable even in those who are not admitted to the hospital during the initial stage of the infection and intensify as per the level of care required during this phase [12]. Recognize and manage persons who have unaddressed or undetected risk factors for CVD in order to avert subsequent cardiovascular incidents resulting from the COVID-19 outbreak [13]. The outbreak of COVID-19 has resulted in a decrease in hospital admissions for all acute cardiovascular illnesses. However, there has been no alteration in hospital mortality rates except for acute aortic dissection, which has seen a rise [14].

In a vast population of unscreened COVID-19 patients across 30 medical facilities in Italy, impaired kidney function, heightened levels of C-reactive protein, and progressed age were notable indicators of mortality during hospitalization. These observations imply that pre-existing conditions, underlying illnesses, and clinical metrics may influence the likelihood of unfavourable outcomes and inpatient fatality in the European region [15].

In an explanatory analysis of 1099 instances of COVID-19, 24.9% of the individuals had concurrent ailments such as hypertension (15%), diabetes (7.4%), and coronary artery disease (2.5%). Aged patients (65 years and above) with concurrent ailments and acute respiratory distress syndrome are at an augmented peril of mortality. Numerous analyses have demonstrated a heightened vulnerability to Middle East respiratory syndrome (MERS)-CoV and human papillomavirus infections in patients with CVD, plausibly due to endothelial dysfunction, metabolic abnormalities, and the escalation of pro-inflammatory cytokines. CVD is a hazard factor for an unfavourable prognosis and significantly amplifies mortality from MERS. Various clinical analyses have demonstrated that CVD is the most prevalent concurrent ailment in patients with COVID-19, and the CVD frequency is elevated in severe and fatal instances [16].

Cardiac insufficiency (CI) is a significant worldwide public health challenge, impacting 64 million individuals globally. The number of hospitalizations due to CI has increased by over three times in the last three decades and is linked to a high mortality rate. It places a substantial financial burden on public healthcare systems and has a noteworthy influence on the well-being of those affected [17].

The latest outbreak has hastened the acceptance of remote medical care in heart health and stimulated the progress of technological innovations like the metaverse. CardioVerse refers to the concept of incorporating the metaverse in cardiac medicine, which has multiple uses such as boosting medical consultations, aiding in heart-related procedures, and reforming medical learning. Despite the probable hindrances in different areas, the usage of unique tokens as safeguarding resources for patient information is emerging as a viable answer [18].

Timely identification of risk factors associated with infectious viral diseases can be crucial in preserving lives by efficiently allocating medical resources and prioritizing susceptible patients during national and global health crises [19]. Timely identification and preemptive measures against illnesses are crucial in averting their aggravation [20]. Managing contagious diseases is a primary concern for public health, and timely identification of infections is crucial to avert outbreaks and global health crises. Scientists are constructing frameworks for timely detection [21].

Knowledge regarding body position, alterations in posture, as well as active and stationary labour is crucial in comprehending the mechanical stresses and ergonomic principles [22]. Ecology and environment greatly rely on biodiversity information; however, various fields must collaborate to handle, exchange, and merge data in disease investigations [23].

CVDs exert a significant weight on the healthcare system, especially in the older population, owing to the presence of various coexisting conditions [24]. Chronic kidney disease (CKD) is the primary reason for mortality in individuals having CKD, where CVD is the primary cause of fatality [25].

Exercise is a crucial non-drug treatment for preventing and treating heart diseases. However, the impact of the length of physical activity on the risk factors related to heart health in grown-ups is not yet clear [26]. The intake of coffee has been demonstrated to have advantageous impacts on metabolic disorders, albeit it could upsurge lipid levels. Additionally, it diminishes the possibility of coronary artery disease, HF, heart arrhythmias, stroke, CVD, and death from all causes. The regular consumption of coffee and tea can be viewed as a component of a salubrious lifestyle and should not be disallowed for patients with CVD [27].

Factors that affect health, known as social determinants of health (SDoH), encompass economic, societal, ecological, and psychological elements. These determinants have a noteworthy effect on the health of individuals with CVD, as well as their outcomes, globally. To achieve health equity and tackle health disparities, SDoH involve determinants related to the framework, physicality, nutrition, and societal environment [28]. The economic condition of an individual is a factor that increases the risk for CVD, and a meagre family income can exacerbate the risk. The government should focus on reducing inequalities and enhancing cardiovascular results in underprivileged groups with low family incomes [29].

The demand for healthcare technology solutions has risen due to the increase in population and changes in lifestyle. Cancer prognosis can determine the likelihood of survival and indicate the seriousness of the illness as it pertains to the patient’s future [30]. The medical industry is experiencing a surge in machine learning (ML) applications as they have the potential to accurately identify patterns in data. This capability can be leveraged to provide accurate diagnosis and prognosis of CVDs, leading to a reduction in misdiagnosis and improved patient care [31].

Forecasting and identification of cardiac ailments pose the greatest hurdles in the field of medicine and rely on facets such as medical evaluations and patient indications. ML methodologies play a pivotal and precise part in the prognosis of heart disease, and technological advancements have facilitated the amalgamation of machine communication with vast data utilities to handle unorganized and rapidly augmenting data [32]. The sole approach to acquiring significant insights in healthcare is through big data, and it is imperative to combine data from diverse origins to discover remedies [33]. The potential of big data to enhance healthcare services and financial returns is immense. Many industries, healthcare included, are making efforts to leverage this potential. By merging bio-medical and healthcare data, contemporary healthcare institutions can bring about a revolution in medical treatment and customized healthcare [34].

The extraction of valuable information from structured human-generated, computer-generated, and sensor data is known as data mining. This involves the collection, cleansing, processing, analysis, visualization, and interpretation of data, using sophisticated learning algorithms to identify patterns and relationships that can be applied in various fields. To achieve more straightforward and comprehensible outcomes, statistical, mathematical, and ML approaches have been employed. Data mining is more than just a task; it encompasses the entire process of gathering, cleaning, processing, analyzing, visualizing, and interpreting data to extract valuable insights [35].

Data analysis is a crucial factor for achievement, as it enables the selection of appropriate data scrutiny methods and the formulation of data-driven products. Nonetheless, businesses frequently encounter a shortage of expertise or time to develop a comprehensive comprehension of data in data analysis. Familiarity with the attributes of the data is vital for devising data-driven products [36].

Unsupervised ML techniques have the potential to uncover risk determinants in patients with intricate clinical conditions like HF, which exhibits indications and manifestations of excess fluid. The worldwide occurrence and frequency of HF have surged, leading to a global outbreak. Novel approaches need to be explored to enhance the management of HF patients [37].

ML algorithms have the potential to enhance the diagnostic and prognostic capabilities of conventional regression methods, however, the outcomes are reliant on the data analysis software utilized [38]. ML techniques are employed for forecasting heart diseases, however, there exists variations in their parameters, aiding physicians in comprehending the information and executing the most suitable techniques [39]. ML is a crucial instrument in public health for recognizing and anticipating communities with higher chances of experiencing health consequences. Thus, it should be incorporated into medical education to direct and decipher scientific investigations [40].

The extraction of significant data from vast quantities of unprocessed information is known as data mining. This technique is utilized in various domains, such as scientific research. Orange employs segment-oriented visual programming to carry out data mining, AI, and inspection tasks. By linking pre-defined or user-provided components known as widgets, work forms are established. The process of constructing a data mining model involves activities such as reading, processing, visualizing, collecting data, and obtaining prediction models [41].

The medical industry is experiencing a transformation in decision-making procedures, thanks to the abundant digital information stored in hospitals, and the implementation of data mining and ML methods. While conventional ML techniques were previously utilized to forecast cancer survival rates, experts are currently transitioning towards deep learning and hybrid approaches to obtain a better understanding of survival prediction [42].

Modern health information systems are distinguished by their ability to rapidly grow and adapt to identify significant health trends and provide timely prevention support. ML-based systems can predict and diagnose heart diseases. Active learning techniques enhance classification accuracy by incorporating expert feedback from users with sparsely labelled data. The label-ranking classifier selection method employs hyperparameters optimized through network search and implements predictive modelling in the cardiac dataset scenario. Experimental evaluations were conducted to measure accuracy and F-score, with and without hyperparameter optimization. The optimized setting prioritized the selection method with respect to the F-score [43].

This study presents a technique for creating data mining models that investigates the performance, classification accuracy, and number of groupings in CVD predictive datasets using the Orange data mining software in unsupervised ML. Orange is a powerful instrument for analyzing and displaying data, identifying data trends, and improving performance. It delivers a user-friendly interface that can be adapted to various domains of research.

### Literature review

The medical sector produces vast quantities of intricate information on patients, hospital assets, sickness diagnoses, digital health records, and medical apparatus. The potential of data mining applications is immense, with some of the most significant applications encompassing forecasting and identification of diseases, evaluating the efficacy of treatments, healthcare operations, prevention of fraud and abuse, customer relationship management, and the medical apparatus industry. An incorrect treatment selection can result in unfavorable consequences, such as patient mortality. Data mining can aid in forecasting and defining diseases within the field [44].

The healthcare sector has made significant progress, resulting in the accumulation of extensive healthcare data, such as electronic health records (EHRs), wearable sensors, and intelligent devices. This data holds undisclosed insights that can aid in making informed decisions. Extracting valuable information requires a thorough search of medical records, and open-source initiatives offer a wealth of data sources for diagnosing and predicting all illnesses [45]. Detecting abnormal sequences plays a crucial role in establishing and safeguarding contemporary health information technology (HIT) systems, ensuring a thorough account of the patient’s condition and occurrences. Nevertheless, this can result in skewed data, intricate interconnections among events in sequences, and diminished complexity [46]. HIT usage poses a challenge in Sub-Saharan Africa, resulting in inadequate patient data. A dependable hospital patient database is crucial for delivering superior healthcare and facilitating seamless communication between healthcare professionals [47].

Valuable information about individual patients and populations is stored in EHRs. The most frequent sources of unstructured EHR data are clinical text and images. Statistical algorithms like natural language processing, radiomics, deep learning, and ML are increasingly being utilized to analyze clinical texts and images. However, explaining and generalizing the outcomes of ML models in healthcare is a crucial and unresolved issue. To enhance the quality and access of unstructured data, developing ML methods that can produce clinically relevant synthetic data and de-identify clinical texts to speed up further research is a potential solution. This is achieved by creating privacy protection technologies such as pseudonymization [48].

Maiga et al. [49] conducted a comparison of ML algorithms for predicting CVDs using a dataset of 70000 medical records. The random forest (RF) model demonstrated impressive results with a classification accuracy of 73%, specificity of 65%, and sensitivity of 80%. These findings have significant implications for the medical industry as they could be utilized to predict the occurrence of CVD.

Peng et al. [50] created an XGBH model for predicting the risk of CVD using significant features extracted from 14832 CVD patients in Shanxi, China. Although it had a slightly lower precision and reduced efficacy in predicting CVD risk, it facilitated timely intervention and economical screening of high-risk patients.

Nouraei et al. [51] utilized three distinct unsupervised ML clustering methodologies on a combined data-set of patients affected by heart failure and preserved ejection fraction. The partitioning around medoids technique recognized six unique groups of patients with varying long-term results or mortality rates, whereas the other two clustering algorithms were subpar.

Detecting anomalies and irregularities in heart rate (HR) and other attributes can aid in comprehending the cause of the disease. The vast quantity of information produced by sensors in portable gadgets has irregularities that necessitate meticulous automation procedures for detection. Several techniques have been suggested to recognize these anomalies [52]. Ripan et al. [53] utilized five ML classification methods to construct prognostic models of results, which were authenticated exploiting customary cardiac datasets. They eliminated abnormalities and employed K-nearest neighbors (KNN), RF, support vector machine (SVM), naïve Bayes, and logistic regression (LR).

The utilization of ML algorithms can aid in the early detection and diagnosis of heart disease, leading to enhanced patient results. Additionally, they can assist patients in managing their condition and daily habits more effectively, ultimately increasing their likelihood of recuperation and survival. This is a positive indication that ML algorithms have the potential to identify illnesses sooner and enhance patient outcomes [54]. Magesh and Swarnalatha [55] proposed a method that utilizes Cleveland heart samples from the University of California, Irvine repository to predict CVD. The precision of the HF classifier was improved by 89.30% through the application of the cluster-based decision tree (DT) learning approach, leading to a significant reduction in the HF error rate from 23.30% to 9.70%.

Shrifan et al. [56] enhanced the accuracy and centroid convergence of k-means clustering through modifications. The newly suggested distance metric surpassed the majority of the literature, leading to an enhancement in the overall clustering accuracy for nine standard multivariate datasets to 80.57%.

The World Health Organization endeavoured to create, assess, and explicate updated models for determining the risk of CVD in low- and middle-income nations. Kaptoge et al. [57] observed significant discrepancies in the projected 10-year risk for a specific risk factor profile among different regions worldwide. After examining data from 79 countries, it was deduced that the percentage of people aged 40-64 years with estimated risk greater than 20% varied greatly, ranging from under 1% in Uganda to over 16% in Egypt.

Nadakinamani et al. [58] proposed an ML-based CVD forecasting system that is extremely precise. The system’s suitability was determined by assessing several metrics, and the random tree model produced excellent results, achieving a 100% accuracy rate, a 0.0011 mean absolute error, a 0.0231 root mean squared error, and a prediction time of only 0.01 s, the fastest of all models tested.

The platform is restricted to supervised ML algorithms, which confine the training dataset to labelled datasets. However, it supports unsupervised learning algorithms, enabling the platform to handle diverse types of training datasets. The selection parameters are set before the training process, which restricts the system options but permits users to modify them post-training to better align with their requirements and enhance efficiency [59]. Aggrawal and Pal [60] suggested a method for identifying mortality in cardiac patients receiving therapy using a sequential feature selection algorithm. To evaluate the accuracy of the selected feature selection (SFS) algorithm against the RF classifier, various ML algorithms such as linear discriminant analysis, RF, gradient boosting classifier (GBC), DT, SVM, and KNN were employed. According to the findings of the experiment, the SFS approach achieved an accuracy of 86.67%.

Ishaq et al. [61] employed nine categorization techniques: DT, AdaBoost, LR, stochastic gradient descent (SGD), RF, GBC, extra tree classifier (ETC), Gaussian naive bayes, and SVM. The issue of imbalanced classes was tackled by utilizing synthetic minority oversampling technique (SMOTE), and the RF was used to identify the most highly-ranked features to train the ML models. The experimental findings demonstrated that ETC outperformed the other models, achieving a SMOTE accuracy score of 0.9262 in predicting the survival of patients suffering from heart disease.

Healthcare experts frequently face difficulties in precisely forecasting heart ailments because of intricate tasks and concealed information, which necessitate contemplation and understanding [62]. Li et al. [63] suggested a ML technology-based system that is both efficient and precise in diagnosing heart ailments. The experimental findings indicate that the feature selection algorithm (FCMIM) proposed by Li et al., with high-level classifier support vectors, is suitable for creating intelligent cardiac detection systems. The diagnostic system (FCMIM-SVM) suggested by Li has demonstrated impressive accuracy in comparison to previously suggested methods and can be conveniently adopted in healthcare for the identification of heart diseases.

The outcomes of Oyeleye et al. [64] experiment demonstrated that by employing forward walking validation and linear regression, the autoregressive intregated moving average model can precisely anticipate the HR for all time spans, while other models are effective for time spans exceeding 1 min. This method of data analysis can be utilized to more accurately predict future HR with the help of accelerometers.

Mohammedqasem et al. [65] created an optimization system based on deep learning to enhance patient classification by processing unbalanced datasets. The system employs SMOTE and a feature-removal algorithm that operates recursively to identify the most efficient features. The experimental predictions demonstrated high consistency and appropriateness, reaching an accuracy level of up to 98% and 97% respectively.

The field of ML holds great potential in enhancing results by identifying prognostic models and categorizing innovative patient subpopulations. AI is permeating our routine activities via promotional algorithms, music and film preferences, and junk mail filtering, but its capacity to access intricate and multidimensional data is just as crucial in the medical domain. However, this has yet to be fully substantiated [66]. AI has the capability to recognize the ideal research specimens, gather supplementary data points, assess continuous data from research participants, and eradicate data-related inaccuracies in overburdened healthcare systems [67]. The significance of AI in healthcare is growing, especially in the examination or anticipatory evaluation of medical information. Hypertensive patients were researched using Spark data analysis as a platform, and AI techniques were employed to pre-analyze the data for inconsistencies, duplication, inadequacy, disturbance, and inaccuracy [68].

Velu et al. [69] suggested utilizing a technique based on ML to anticipate liver complications by analyzing the outcomes of liver function tests that were conducted during medical check-ups. The system encompasses an interface that medical professionals can utilize to obtain patient data. The patients’ liver function test outcomes were assessed to identify whether they had liver disease by examining the blood levels of enzymes and proteins that are specific to liver function tests.

The use of AI in electrocardiography (ECG) is a prime example of how AI is transforming cardiovascular medicine. Advanced AI techniques, including convolutional neural networks using deep learning, have made it possible to interpret ECGs quickly and accurately, similar to how humans would. This has allowed for the detection of signals and patterns that would have otherwise gone unnoticed by human interpreters. By utilizing extensive digital ECGs that come with comprehensive clinical data, AI models have been developed to identify left ventricular dysfunction, silent atrial fibrillation (previously undetected and asymptomatic), hypertrophic cardiomyopathy, and other phenotypes such as age, sex, and race. As mobile and wearable ECG technologies become increasingly available, the clinical and population-level implications of AI-based ECG phenotyping are still unfolding [70].

The gaps in this study compared to existing or previous research are as follows:

- The use of Orange data mining software for unsupervised ML clustering of CVD datasets has not been explored before.
- The topic of achieving optimal model analysis and classification accuracy across all classification types has seldom been explored.
- Investigations that strive to identify the quantity of clusters utilizing diverse clustering techniques within a single dataset are infrequent.

This research centered on utilizing data mining through the Orange data mining software to examine the precision of classification, number of clusters, and overall performance of CVD prognostic datasets in unsupervised ML, drawing from insights gleaned from prior scientific literature reviews.

This paper is organized as follows: Methods section outlines the research methodology, Results and discussion section presents the results and discussion, Conclusions section summarizes the research conclusions and provides suggestions for future studies of unsupervised ML in the CVD dataset.

## Methods

**Fig. 3:**
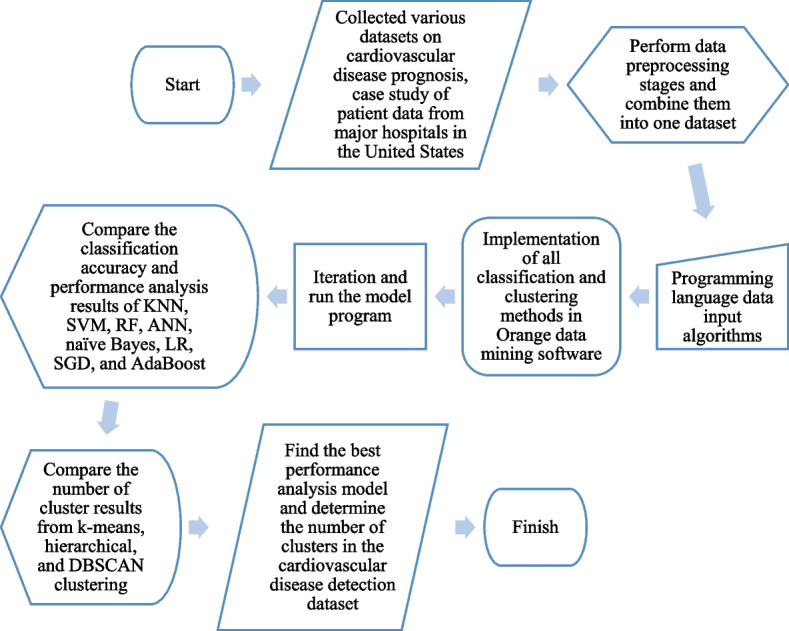
Research flowchart

Figure 3 presents a flowchart of the research stages.

For this research, we utilized diverse datasets on CVD prognosis in a case study of patient information from prominent hospitals in the United States. The participants were individuals aged between 28-77 years. We procured varied datasets from Kaggle, which assembles public information from websites, like frequent visitors, without compromising personal data. The data comprised observational findings of 918 patients from one of the most notable hospitals in the United States. The datasets had 18 characteristics, out of which 2 were categorical and 16 were numerical. The clinical parameters that were available in the dataset (18 attributes) included age, gender, resting blood pressure, maximum HR, old peak, creatine phosphokinase, ejection fraction, platelet count, serum creatinine, serum sodium, time, systolic and diastolic blood pressure, HR (bpm), cholesterol level, low density lipoprotein (LDL) level, high density lipoprotein (HDL) level, and CVD prognosis.

The initial stage of data processing employs a tool for imputation that calculates the average frequency of missing data attributes. After that, a range of classification techniques were employed to model parameters, including KNN, SVM, RF, artificial neural network (ANN), naïve Bayes, LR, SGD, and AdaBoost, to identify the most effective performance analysis and assess classification accuracy. Subsequently, we utilized various unsupervised ML clustering methods, such as k-means, hierarchical, and density-based spatial clustering of applications with noise (DBSCAN) clustering, to determine the number of clusters for CVD patients. The Orange data mining software was utilized for all analyses.

## Results and discussion

### Performance analysis and classification accuracy on Cardiovascular Disease Prognostic datasets

**Fig. 4:**
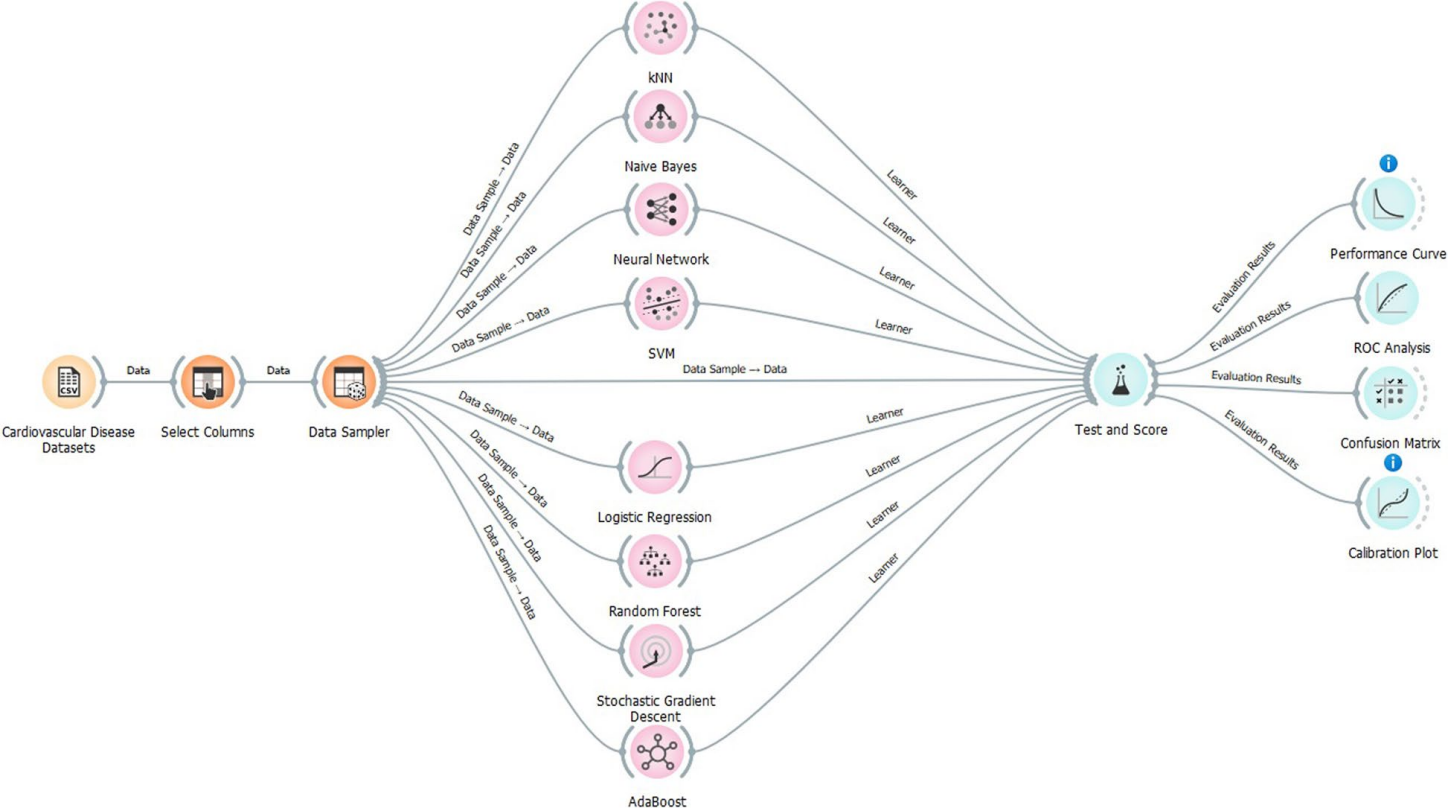
Classification techniques used to classify Cardiovascular Disease Prognostic datasets

Figure 4 shows the overall layout view of the classification techniques on Cardiovascular Disease Prognostic datasets.

**Fig. 5:**
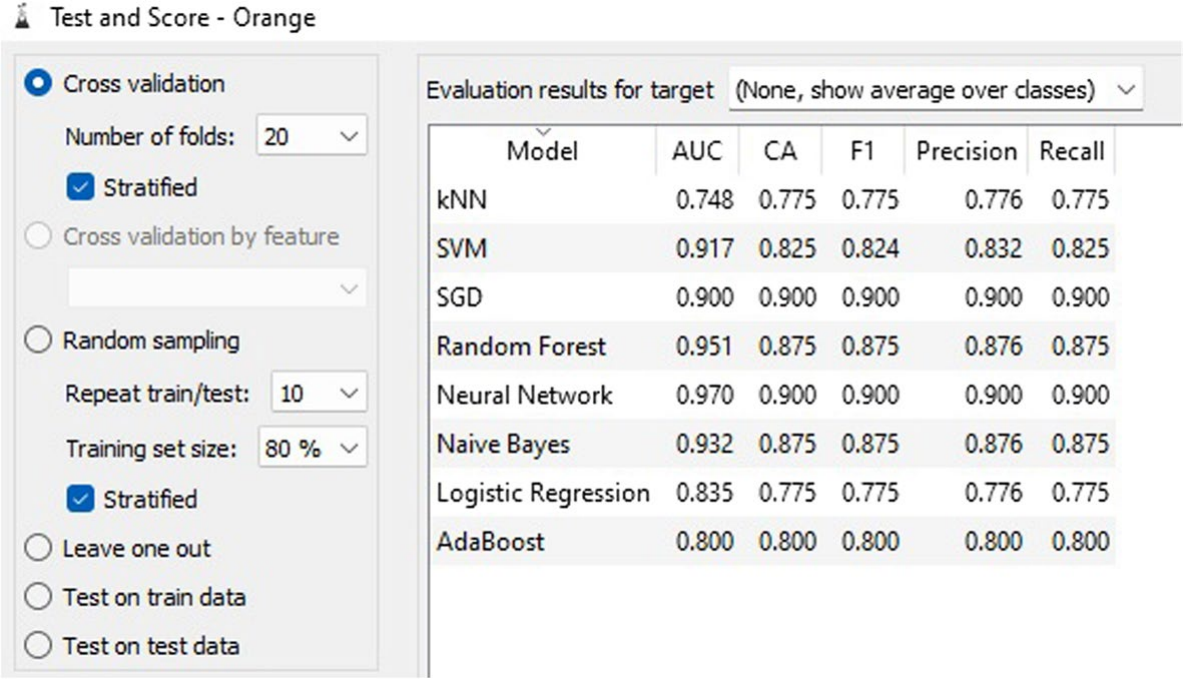
Test and score results

Based on the outcomes and evaluations presented in Fig. 5, it is evident that SGD and ANN are the most efficient techniques for categorizing CVD predictive information.

**Fig. 6:**
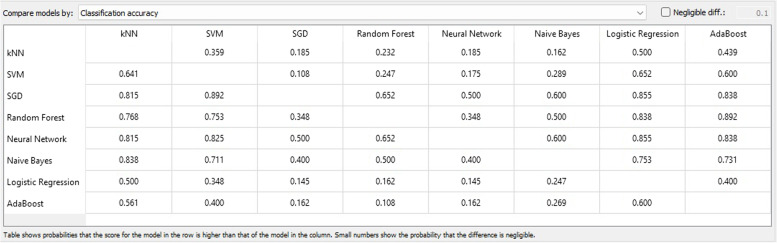
Classification accuracy matrix

The accuracy of classification refers to the percentage of correctly classified instances, whereas the accuracy of words pertains to the degree of proximity between a group of measurements and their actual values. The accuracy matrix for classification can be observed in Fig. 6.

**Fig. 7:**
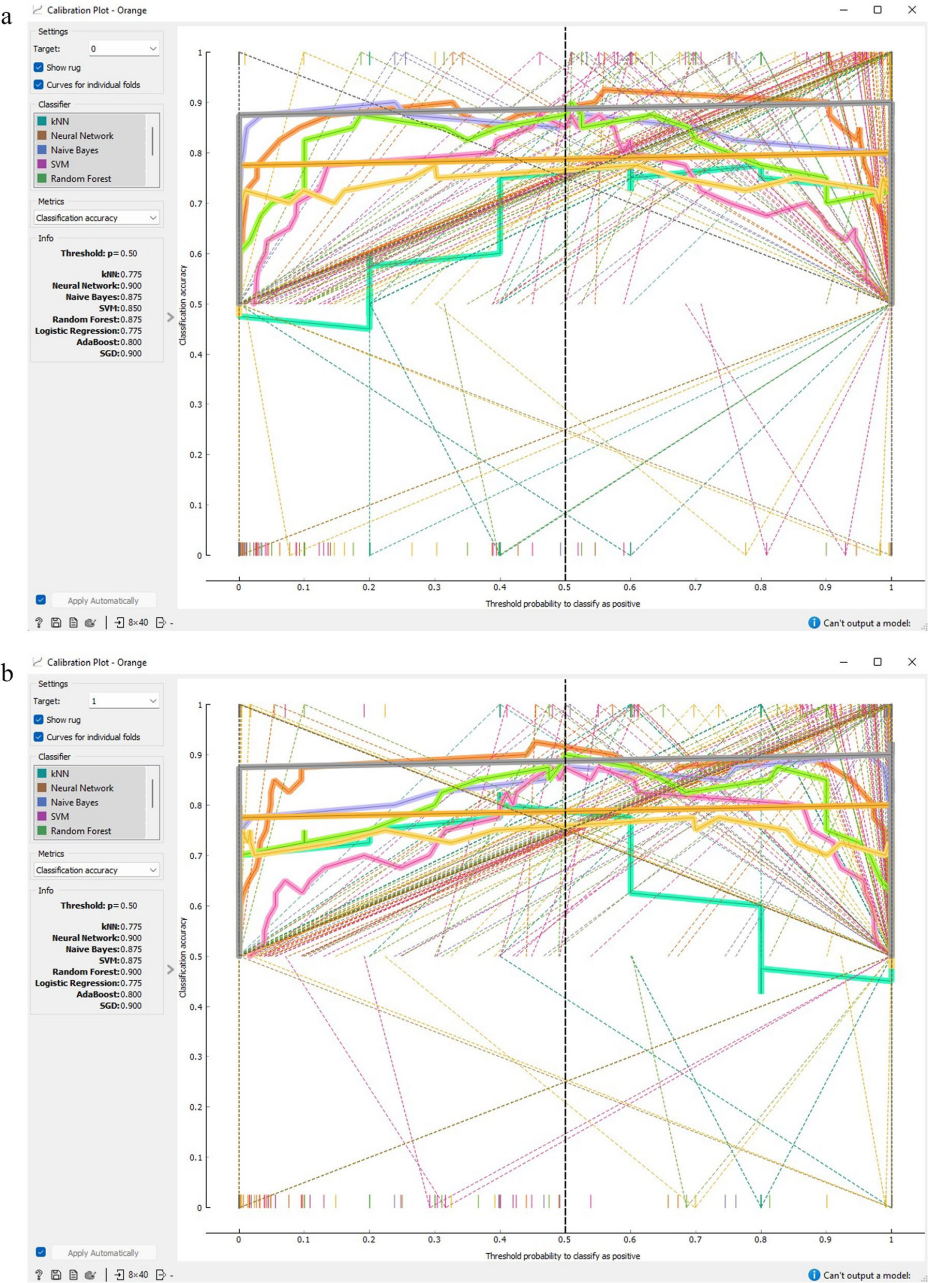
Calibration plot based on classification accuracy CVD prognostic. **a** target = 0; **b** target = 1

Figure 7 demonstrates that SGD and ANN models outperform alternative methods of classification with regards to prognostic data for CVD. The calibration graph charts the anticipated probabilities of the classifier in relation to class probabilities and can serve as a means of verifying if the classifier is excessively hopeful or despondent. Additionally, the tool can exhibit a calibrated model where the user can set their own probability threshold.

**Fig. 8:**
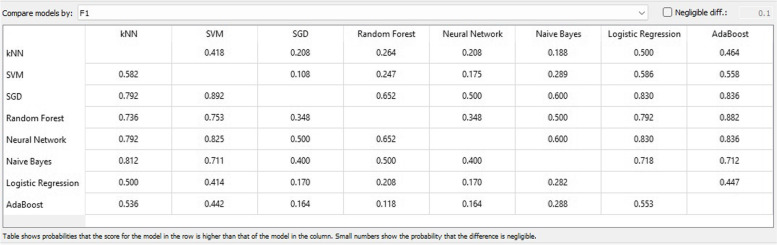
F1 score matrix

Figure 8 displays the F1 score chart which links the models of the attribute categorization methodology. This is a harmonic assessment of precision and recall, signifying both precision and recall in a single measure. The maximum attainable score was 1, indicating impeccable precision and recall, while the minimum was 0.

**Fig. 9:**
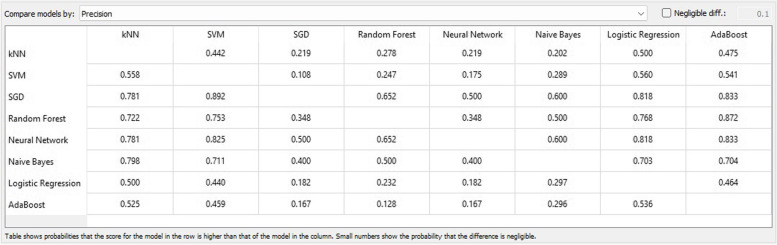
Precision matrix

**Fig. 10:**
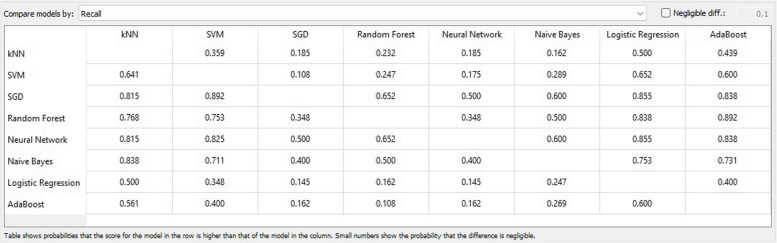
Recall matrix

In the fields of ML, object detection, classification, pattern recognition, and information retrieval, precision and recall serve as performance metrics for data extracted from a sample space, corpus, or collection. Precision, also referred to as positive predictive value, represents the ratio of retrieved instances that are relevant, as depicted in Fig. 9. Sensitivity, or recall, represents the ratio of relevant instances that are retrieved. Consequently, both precision and recall are founded on relevance, as illustrated in Fig. 10.

**Fig. 11:**
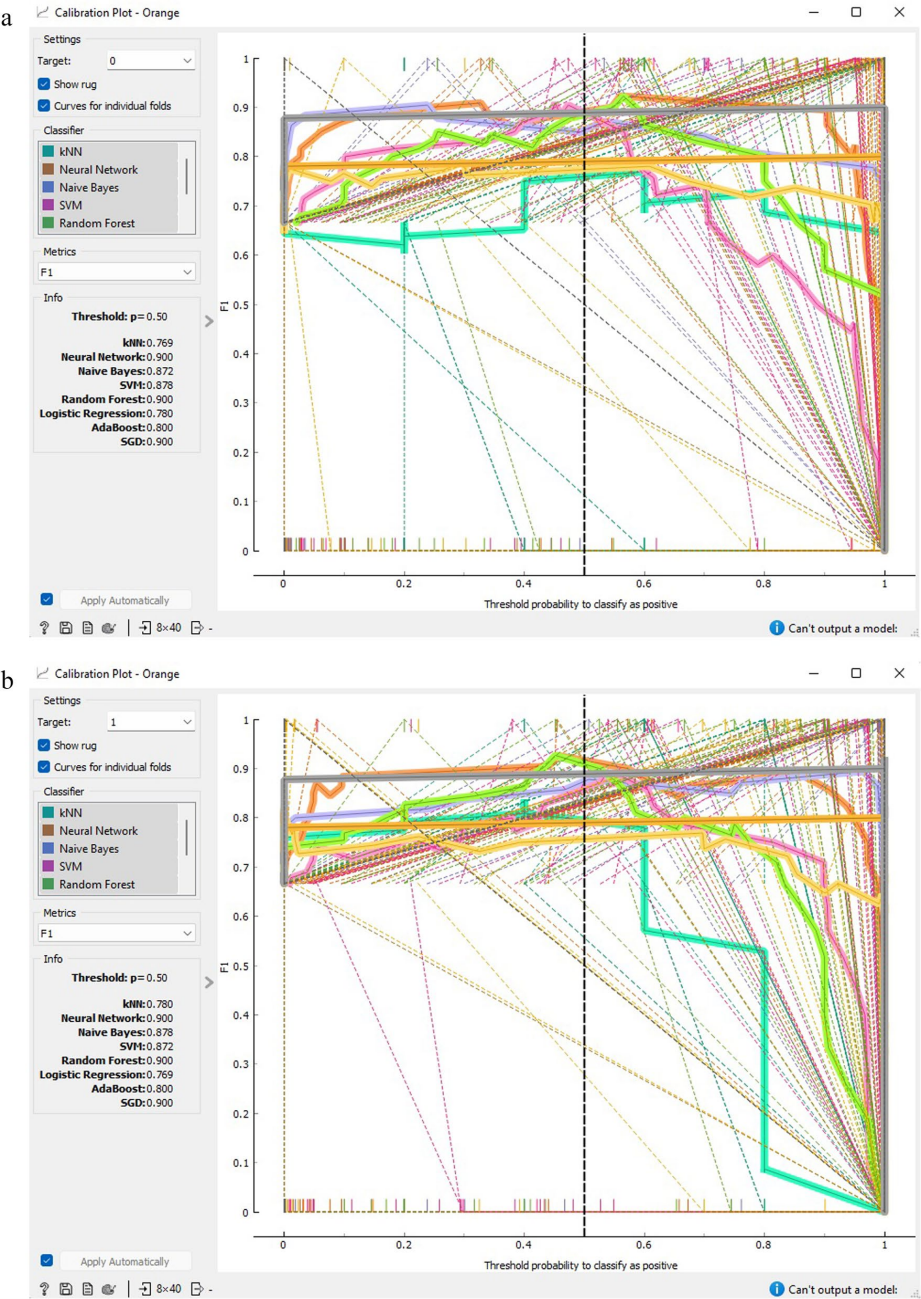
Calibration plot based on F1 score CVD prognostic. **a** target = 0;** b** target = 1

**Fig. 12:**
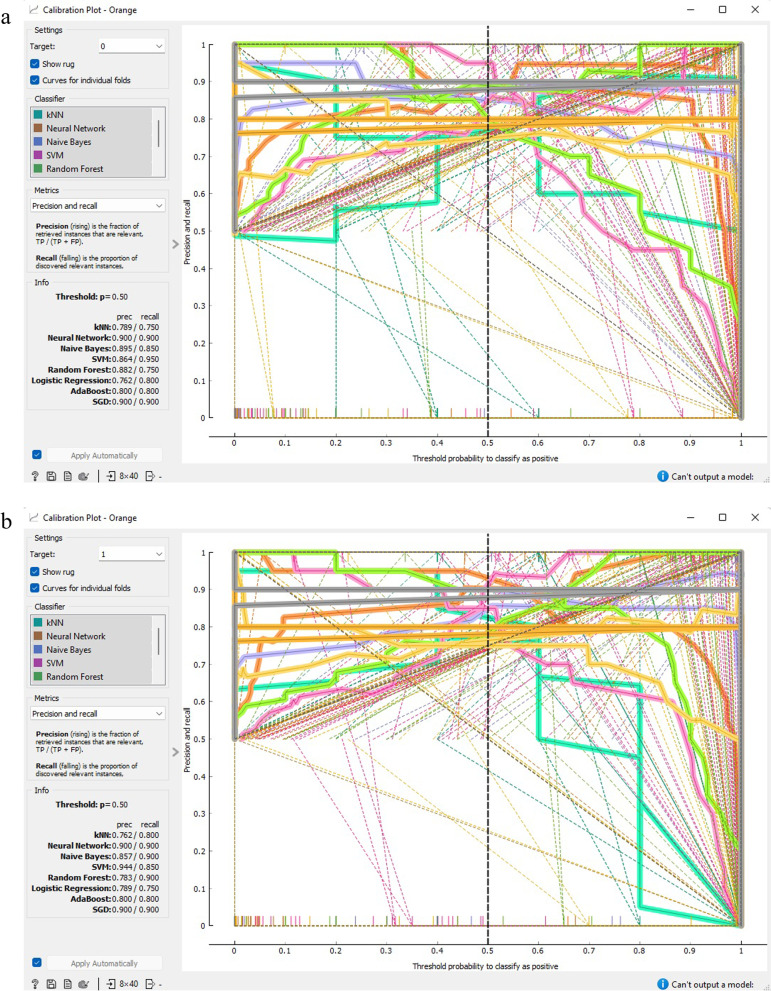
Calibration plot based on precision and recall CVD prognostic. **a** target = 0; **b** target = 1

Figures 11 and 12 demonstrate that both SGD and ANN models outperform alternative classification techniques for prognostic data related to CVD. The calibration curve depicts the anticipated probabilities of the classifier in relation to the class probabilities, enabling the assessment of whether the classifier is overly optimistic or pessimistic. The tool also presents a calibrated model that allows the user to establish their own probability threshold.

**Fig. 13:**
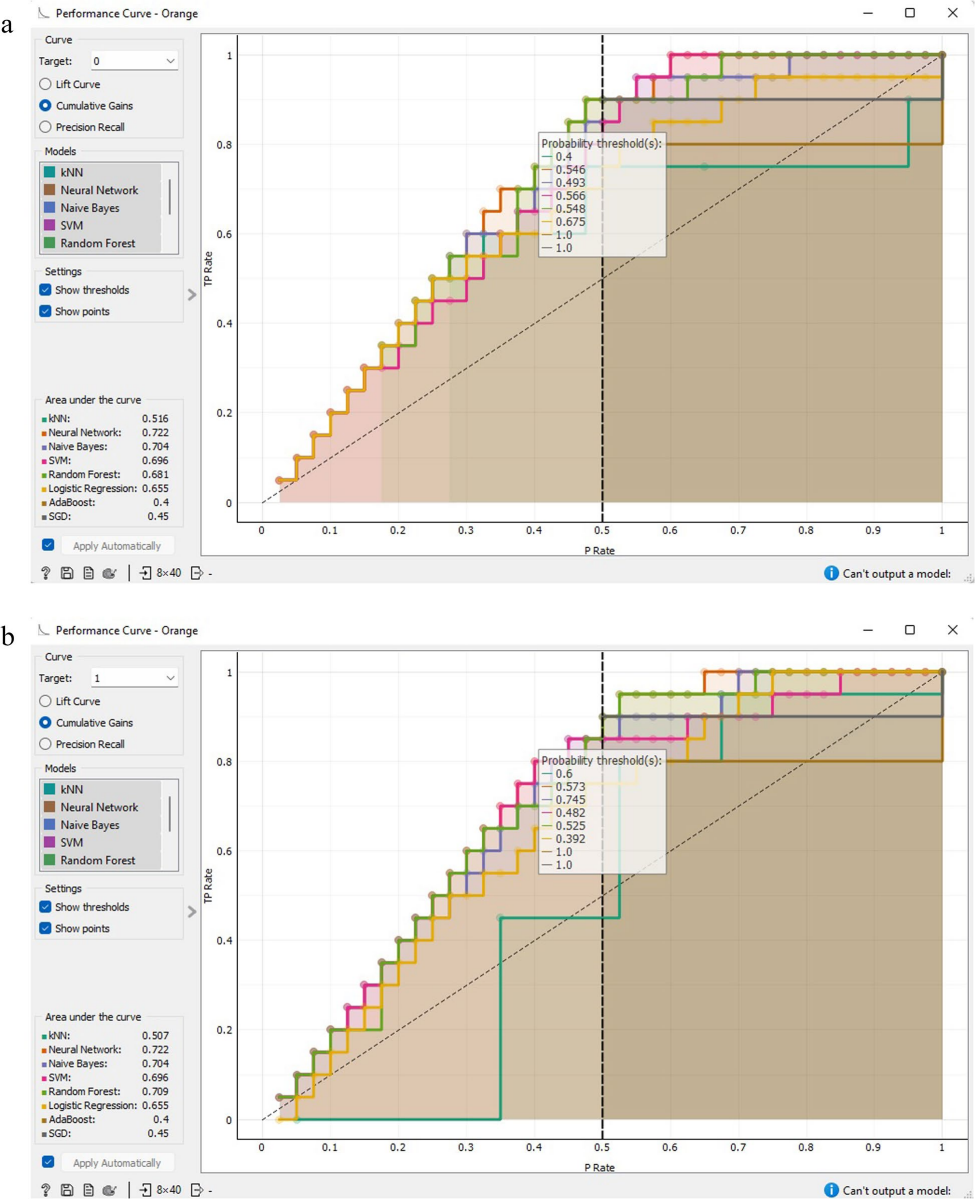
Performance curve analysis CVD prognostic. **a** target = 0; **b** target = 1

Figure 13 demonstrates that the ANN framework exhibited the most optimal performance for categorizing CVD prognostic data. The performance graph represents the ratio of accurate positive data instances in comparison to the classifier’s threshold, while the cumulative return diagram showcases the correlation between actual positive cases and the support. The greater the region between the curve and the baseline (dashed line), the more exceptional the model.

### Determining the number of clusters on Cardiovascular Disease Prognostic datasets

**Fig. 14:**
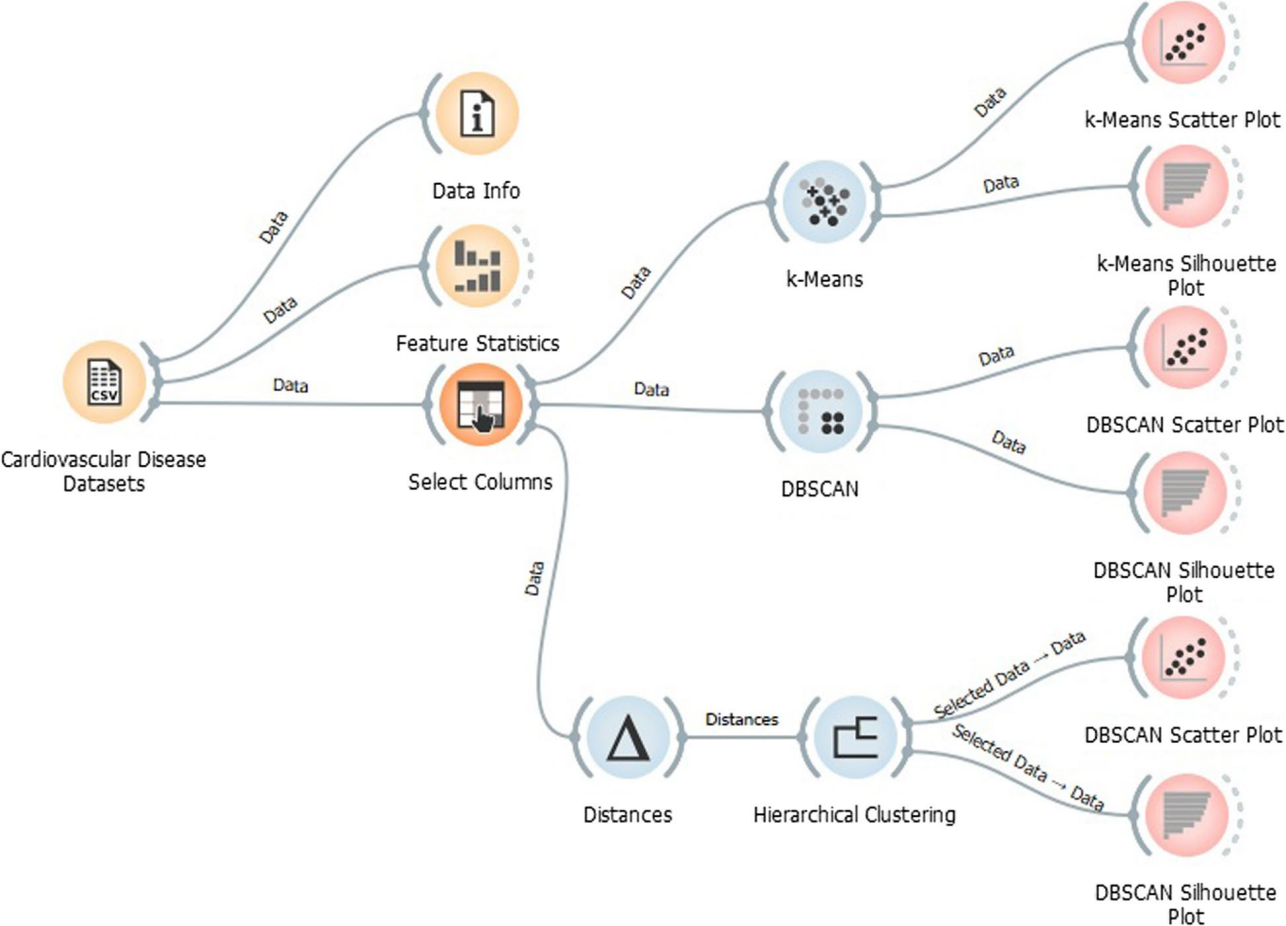
Clustering techniques are used to analyze Cardiovascular Disease Prognostic datasets

Figure 14 shows an overall layout view of the clustering techniques on Cardiovascular Disease Prognostic datasets.

#### (1) K-means clustering on Cardiovascular Disease Prognostic datasets

**Fig. 15:**
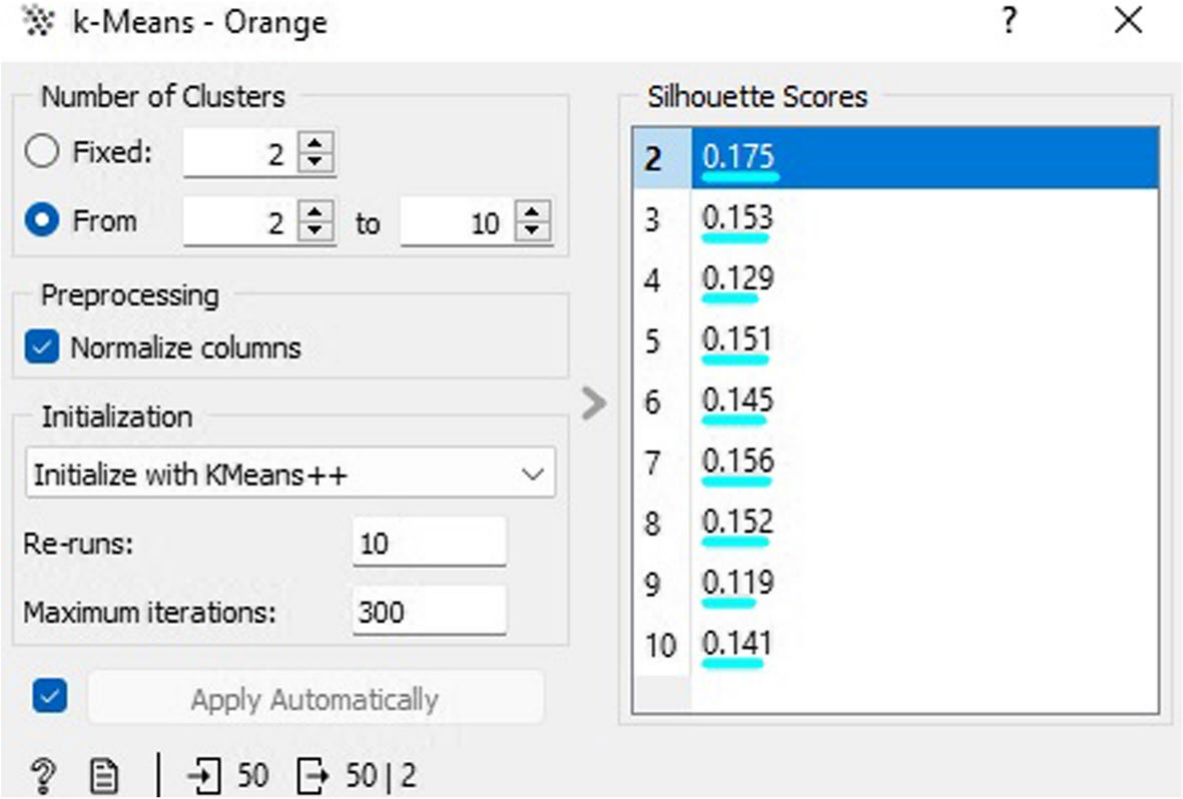
K-means clustering

In the illustration depicted as Fig. 15, the CVD prognostic dataset is partitioned into two clusters using the k-means cluster technique, with a silhouette score of 0.175. The widget algorithm utilizes k-means clustering to process the data and generates an updated dataset that includes the cluster label as a meta-attribute. Additionally, the widget presents the silhouette points of the group outcomes for various k values. A higher silhouette score indicates superior grouping.

**Fig. 16:**
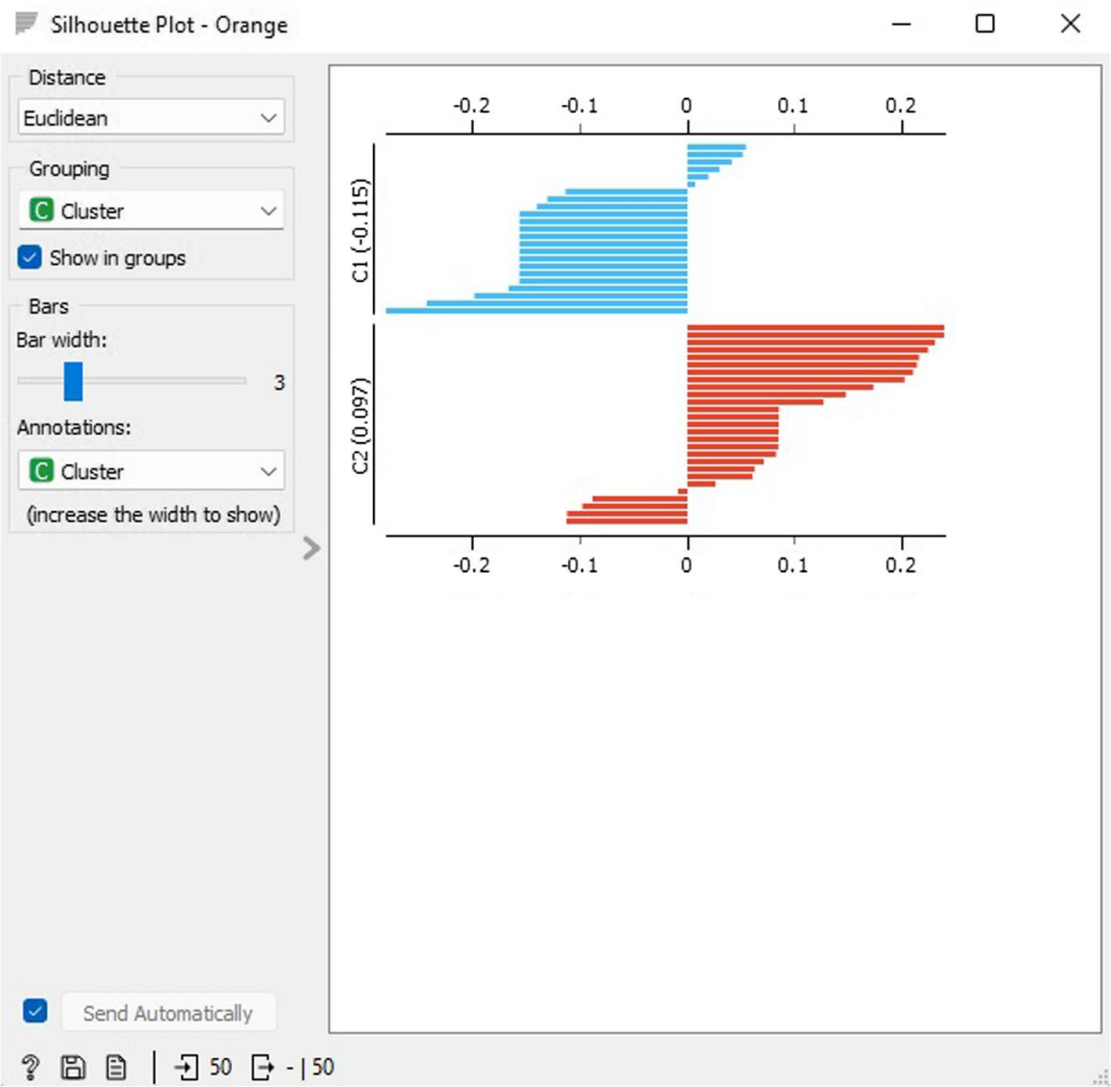
K-means clustering silhouette scores

Figure 16 displays the k-means clustering silhouette outcomes for two groupings. Cluster 1 (C1) has an average value of -0.115 and cluster 2 (C2) has an average value of 0.097. The Silhouette Graph widget presents a pictorial representation of the uniformity of data groupings and enables users to visually evaluate the grouping quality. The silhouette value signifies how comparable an item is to its grouping in comparison to other groupings, and events with a silhouette value close to 1 suggest that the data point is in proximity to the center of the grouping, while events with a silhouette value close to 0 are situated at the boundary of the two groupings.

**Fig. 17:**
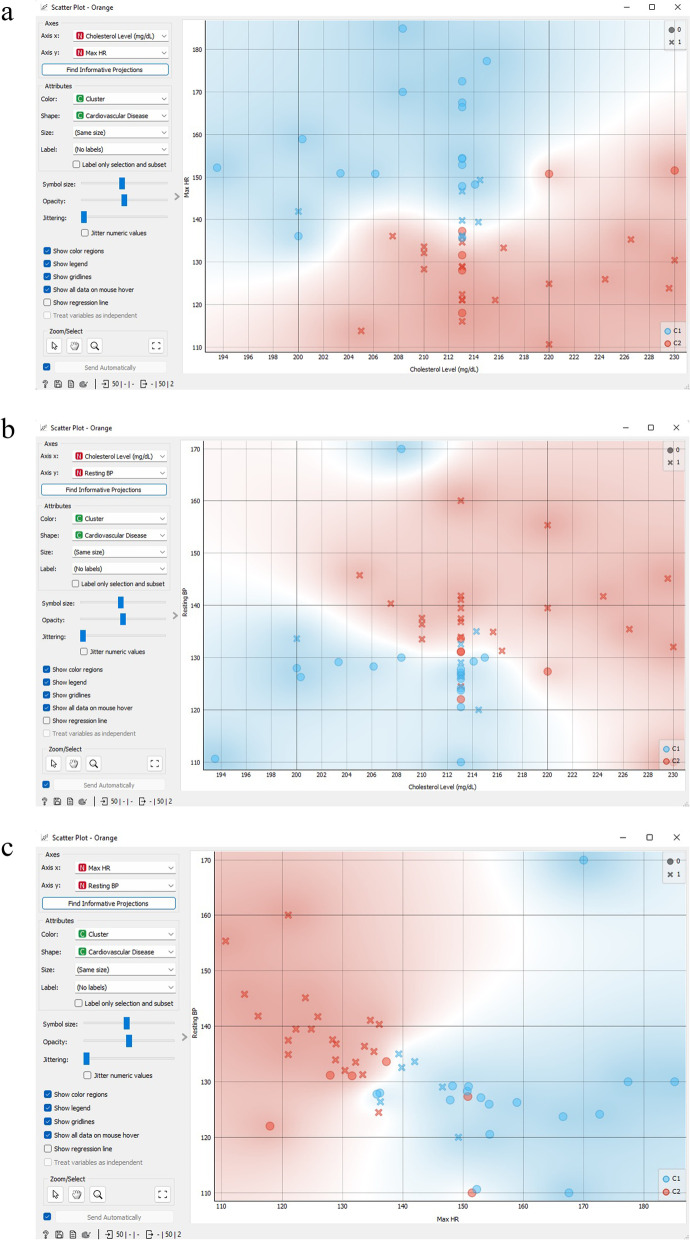
Scatter plot of k-means cluster analysis.** a** K-means clustering scatter plot correlations between cholesterol level and maximum HR attributes; **b** K-means clustering scatter plot correlations between cholesterol level and resting blood pressure attributes; **c** K-means clustering scatter plot correlations between maximum HR and resting blood pressure attributes

Figure 17 exhibits a scatter plot of k-means cluster analysis that depicts the correlation among cholesterol level, maximum HR, and resting blood pressure. The scatter-plot tool showcases a two-dimensional scatter, where information is exhibited as a set of dots with *x*-axis and *y*-axis attribute values. On the widget’s left-hand side, several chart features, including dot hue, magnitude and shape, axis headings, maximum dot size, and jitter, can be modified.

#### (2) Hierarchical clustering on Cardiovascular Disease Prognostic datasets

**Fig. 18:**
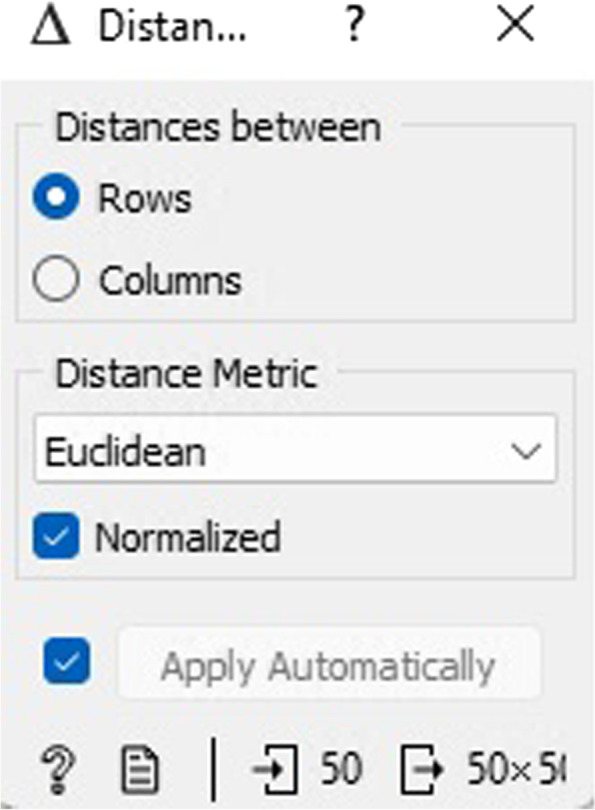
Distances

In the CVD prognostic dataset, Fig. 18 illustrates the normalized distances between rows and columns. The objective of normalization was to guarantee impartial treatment of individual attributes, and it was executed per column.

**Fig. 19:**
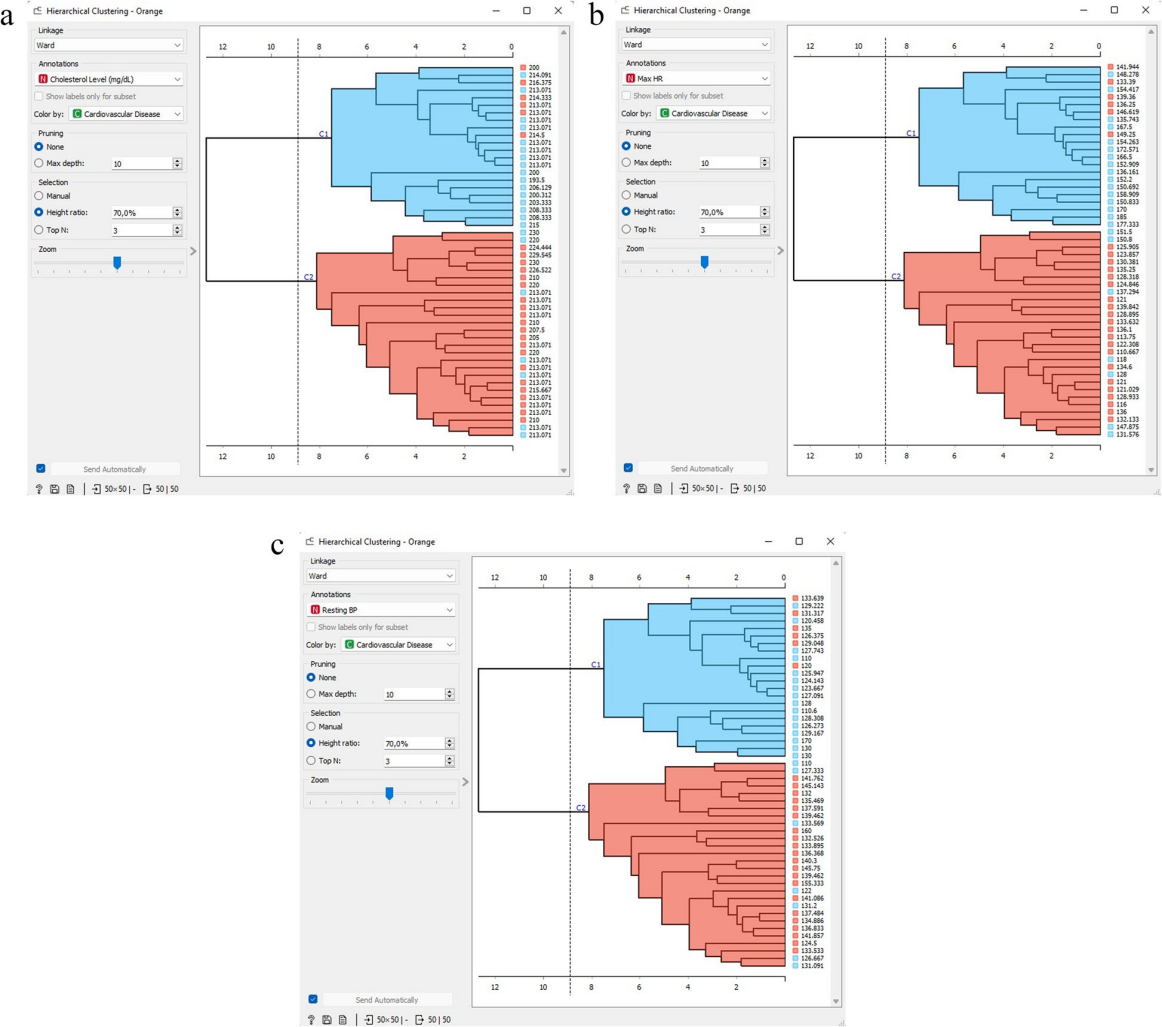
Hierarchical clustering. **a** Hierarchical clustering based on the cholesterol level attribute; **b** Hierarchical clustering based on the maximum HR attribute; **c** Hierarchical clustering based on the resting blood pressure attribute

Figure 19 depicts the outcomes of the hierarchical clustering, which were segregated into two clusters. The distance tool computes the hierarchical clustering of diverse object categories from the distance array and exhibits the associated dendrogram.

**Fig. 20:**
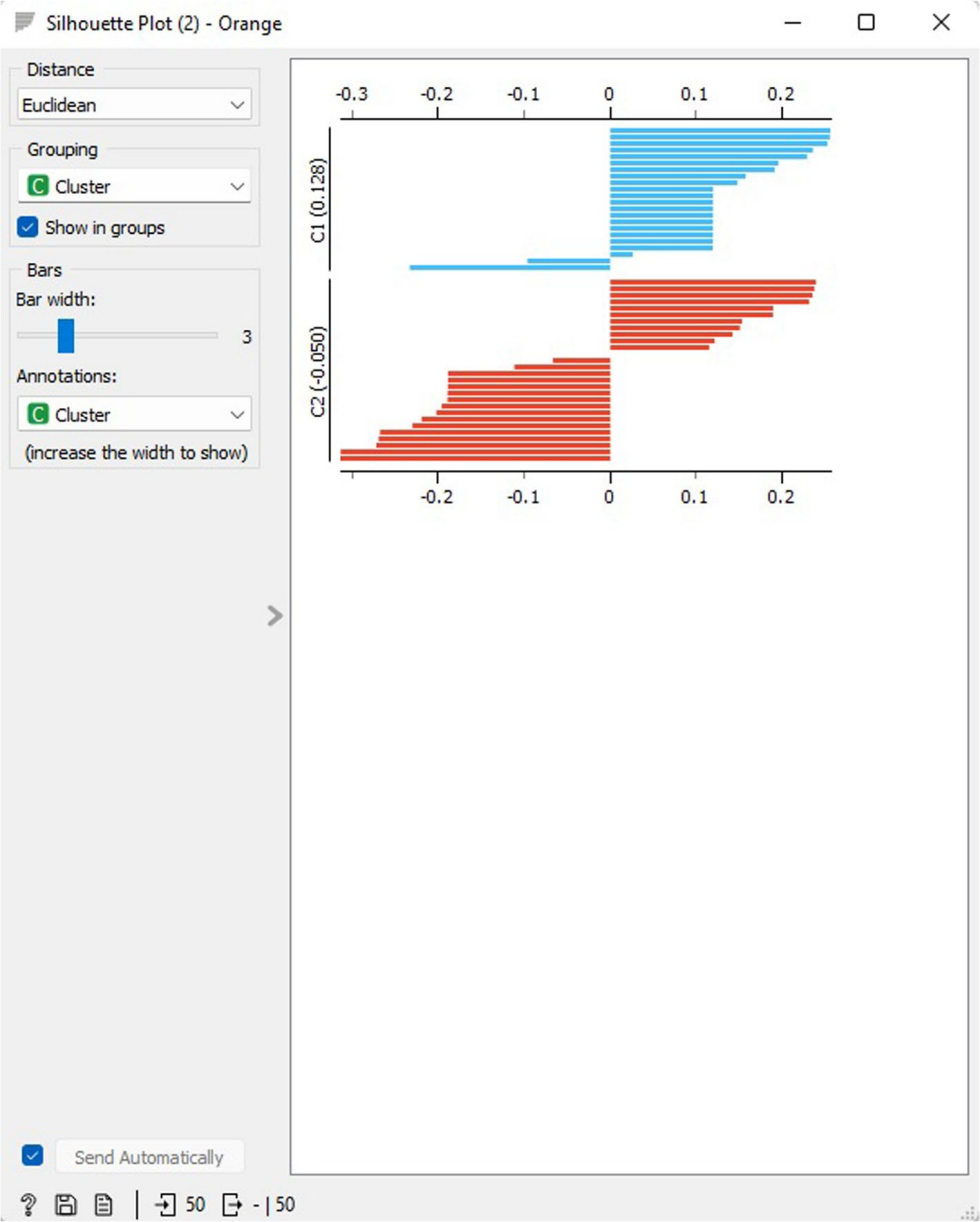
Hierarchical clustering silhouette scores

The outcomes of the hierarchical cluster silhouette analysis for two clusters are presented in Fig. 20, with C1 exhibiting an average score of 0.128 and C2 exhibiting an average score of -0.050. The Silhouette Plot widget enables users to assess the quality of the data clusters visually and depicts the consistency of the clusters. The silhouette score indicates how comparable an object is to its cluster in contrast to other clusters, and objects with a silhouette score near 1 suggest that the data point is located in the center of the cluster, while objects with a silhouette score near 0 are found at the boundary of the two clusters.

**Fig. 21:**
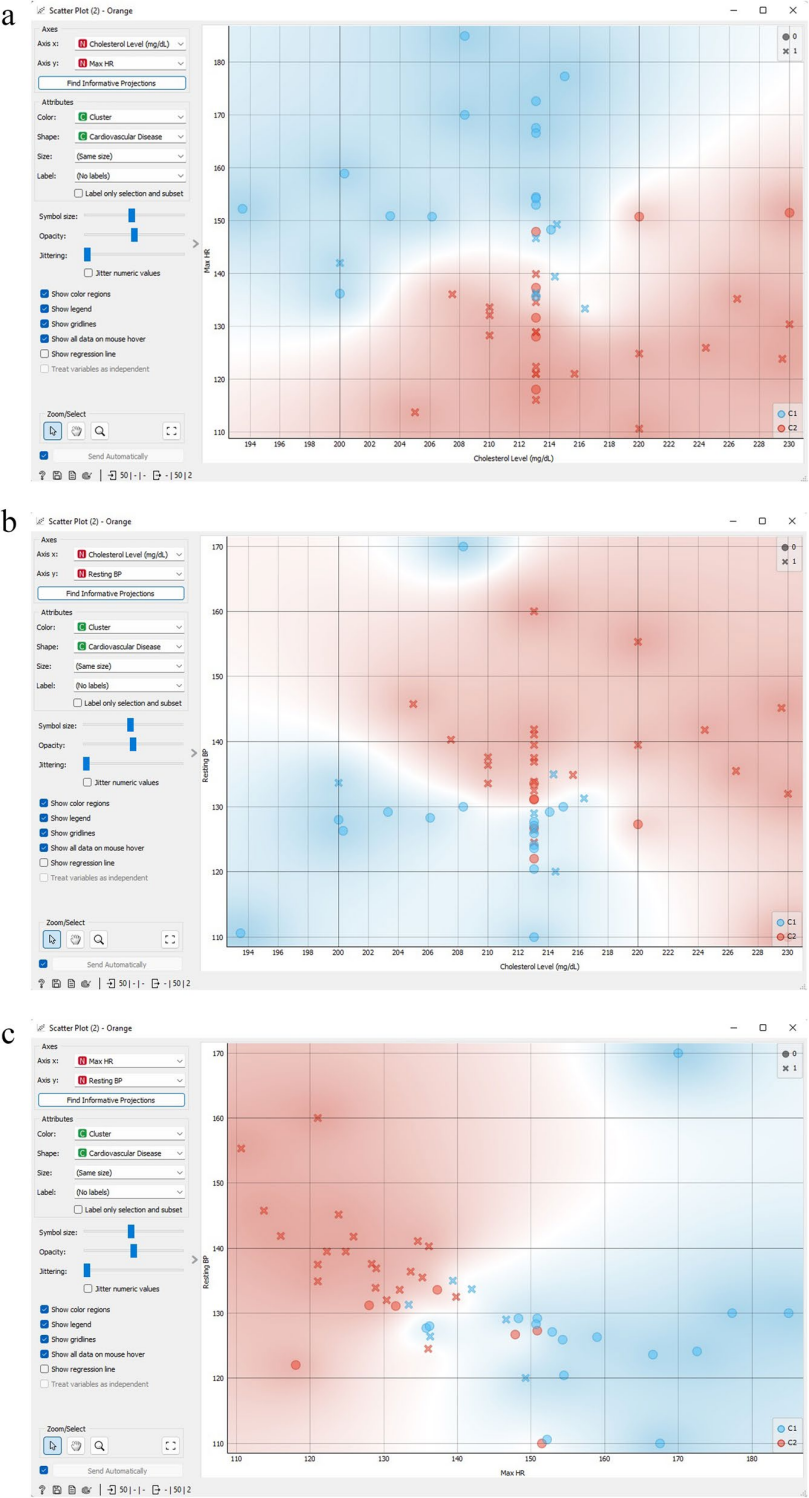
Hierarchical cluster scatter diagram that illustrates the correlation between cholesterol level, maximum HR, and resting blood pressure. **a** Hierarchical clustering scatter plot correlations between cholesterol level and maximum HR attributes; **b** Hierarchical clustering scatter plot correlations between cholesterol level and resting blood pressure attributes; **c** Hierarchical clustering scatter plot correlations between maximum HR and resting blood pressure attributes

Figure 21 exhibits a hierarchical cluster scatter diagram that illustrates the correlation between cholesterol level, maximum HR, and resting blood pressure. The scatter-plot gadget presents a two-dimensional scatter, where the data is portrayed as a set of dots with *x*-axis and *y*-axis characteristic values. On the widget’s left-hand side, different chart features, such as point hue, magnitude and form, axis headings, maximum point size, and jitter, can be modified.

#### (3) DBSCAN clustering on Cardiovascular Disease Prognostic datasets

**Fig. 22:**
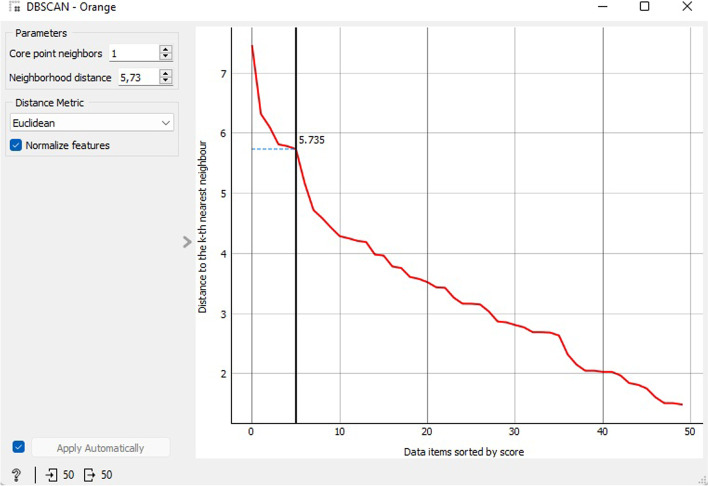
DBSCAN clustering

Figure 22 illustrates the results obtained from the application of DBSCAN. The optimal number of clusters for the CVD prognostic datasets under DBSCAN was segregated into 3 clusters, where the core point neighbors were 1, neighborhood distance was 5.73, and the distance metric was Euclidean. The widget utilizes the DBSCAN clustering algorithm on the data, resulting in a fresh dataset with group identities as meta-attributes. Additionally, it exhibits an ordered chart depicting the k-th nearest neighbor distances, provided the k-values pertain to the core point neighbors. The chart exhibits the distance to the k-th nearest neighbor, ascertained by selecting the core point’s neighborhood. The correct inclusive range can be chosen by shifting the black slider to the left or right.

**Fig. 23:**
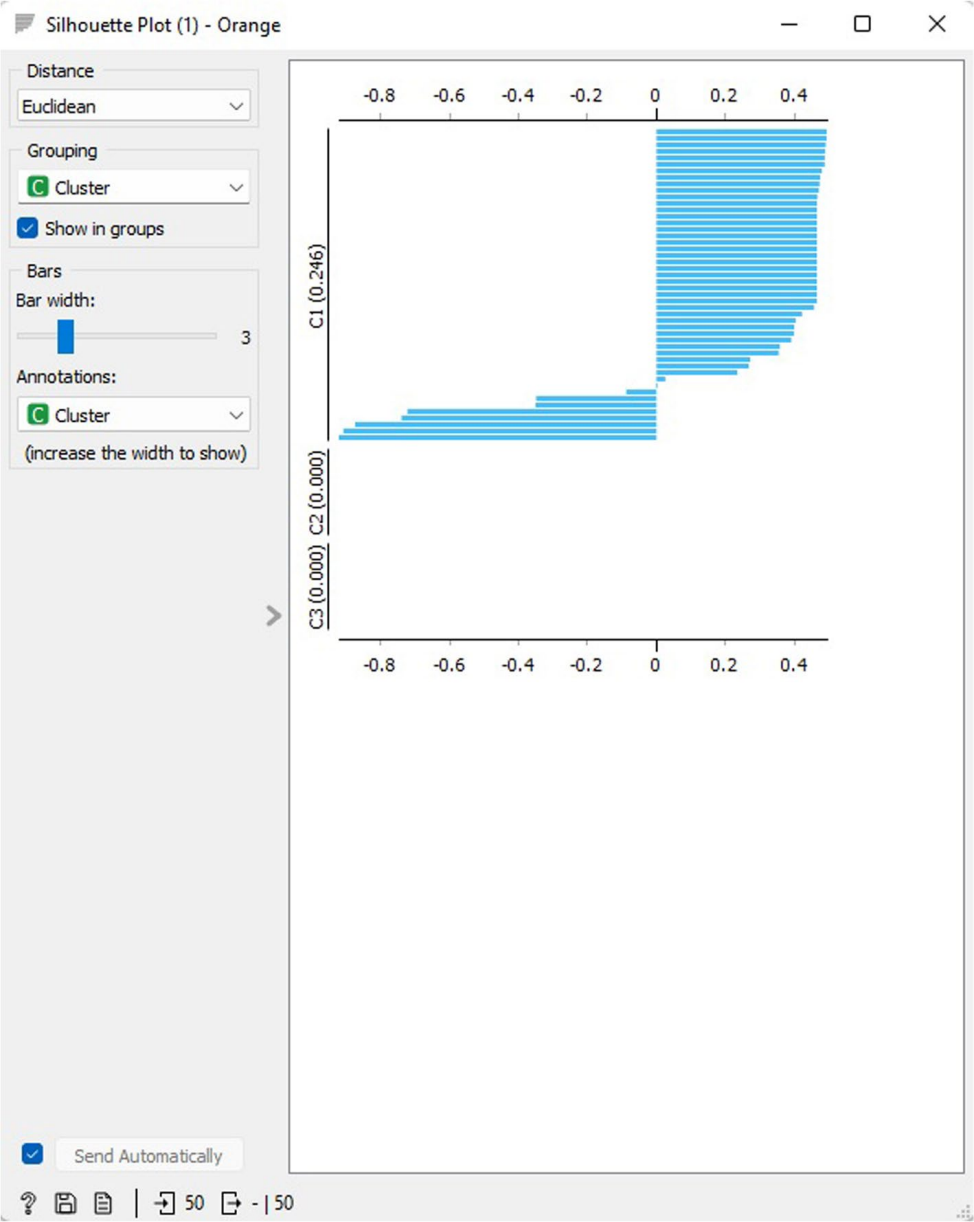
DBSCAN clustering silhouette scores

The DBSCAN cluster silhouette results for 3 clusters are presented in Fig. 23. C1 displays an average score of 0.246, while C2 and C3 both have an average score of 0.000. The Silhouette Plot widget offers a visual representation of the consistency of data clusters, enabling users to evaluate the quality of the clusters. The silhouette score indicates the similarity between an object and its cluster in comparison to other clusters. Scores close to 1 suggest that the data event is positioned close to the center of the cluster, whereas scores close to 0 indicate that the data event is situated at the border of the three clusters.

**Fig. 24:**
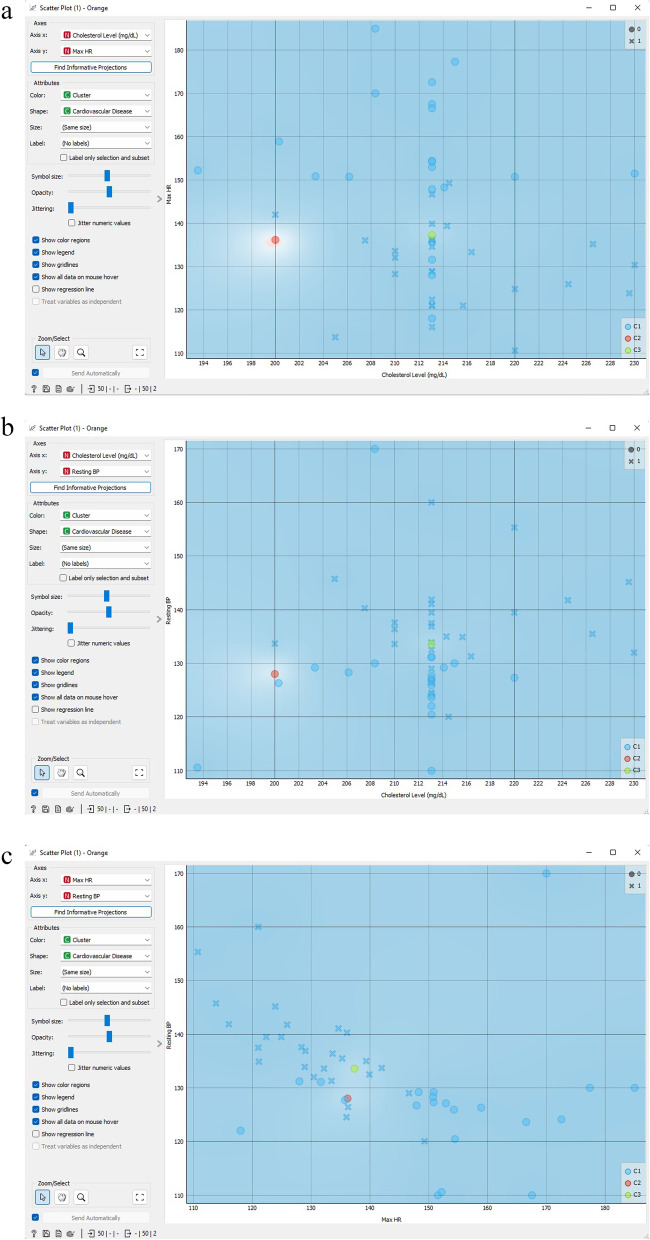
The scatter plot of DBSCAN cluster that demonstrates the correlation among cholesterol level, maximum HR, and resting blood pressure.** a** DBSCAN clustering scatter plot correlations between cholesterol level and maximum HR attributes; **b** DBSCAN clustering scatter plot correlations between cholesterol level and resting blood pressure attributes; **c** DBSCAN clustering scatter plot correlations between maximum HR and resting blood pressure attributes

Figure 24 presents the scatter plot of DBSCAN cluster that demonstrates the correlation among cholesterol level, maximum HR, and resting blood pressure. The scatter-plot tool exhibits a scatter with two dimensions, where the data is exhibited as a group of points with *x*-axis and *y*-axis characteristic values. The widget’s left side allows the customization of different chart characteristics, including point color, size, and shape, axis headings, maximum point size, and jitter.

Table 1 indicates that the CVD predictive datasets can be categorized into two groups, determined by the outcomes of various clustering techniques, including k-means clustering and Hierarchical clustering.

**Table 1:** Comparative results of k-means, hierarchical, and DBSCAN clustering in the CVD prognostic datasets

**Clustering methods**	K-means clustering	Hierarchical clustering	DBSCAN clustering
The best number of clusters	2	2	3
Distance metric	Euclidean	Euclidean	Euclidean
Silhouette scores	C1 = -0.115 C2 = 0.097	C1 = 0.128 C2 = -0.050	C1 = 0.246 C2 = 0.000 C3 = 0.000

## Conclusions

This research used various datasets on CVD prognosis in a case study of patient information from prominent hospitals in the United States. The participants were individuals aged between 28-77 years. We procured varied datasets from Kaggle, which assembles public information from websites, like frequent visitors, without compromising personal data. The data comprised observational findings of 918 patients from one of the most notable hospitals in the United States. The datasets had 18 characteristics, out of which 2 were categorical and 16 were numerical. The clinical parameters that were available in the dataset (18 attributes) included age, gender, resting blood pressure, maximum HR, old peak, creatine phosphokinase, ejection fraction, platelet count, serum creatinine, serum sodium, time, systolic and diastolic blood pressure, HR, cholesterol level, LDL level, HDL level, and CVD prognosis.

The initial stage of data processing employs a tool for imputation that calculates the average frequency of missing data attributes. After that, a range of classification techniques were employed to model parameters, including KNN, SVM, RF, ANN, naïve Bayes, LR, SGD, and AdaBoost, to identify the most effective performance analysis and assess classification accuracy. Subsequently, we utilized various unsupervised ML clustering methods, such as k-means, hierarchical, and DBSCAN clustering, to determine the number of clusters for CVD patients. The Orange data mining software was utilized for all analyses.

The results showed that the most outstanding performance analysis and classification accuracy for CVD prognosis datasets were observed with SGD and ANN. The CVD prognosis datasets were able to be segregated into two clusters through clustering techniques like k-means and hierarchical clustering. The precision of the suggested model in determining the diagnostic model is crucial for the accuracy of CVD prognosis. The better the model’s accuracy, the more reliable it becomes in predicting the patients who are susceptible to CVD.

Prognostic systems for CVDs are valuable in the upkeep and surveillance of patient populations, as well as in the reduction of mortality rates. These systems can serve as a significant tool in raising awareness about personal health and in the early detection and prevention of CVDs. The precision of CVDs prognostic diagnosis relies on the model’s precision in determining the diagnostic model. Therefore, the more precise the model, the more accurate the prediction of patients who may be at risk of developing CVDs.

In this context, we suggest concepts for additional investigation of an unsupervised ML CVDs prognosis dataset.

- Future researchers can explore alternative distance measures employed in different clustering techniques, like Fuzzy c-means and k-medoids clustering, and enhance the operational efficiency of modified k-means by diminishing the time complexity of Cardiovascular Disease Prognostic Rules.
- Future researchers ought to employ a metaheuristic-based feature selection approach that takes features as input and organizes the original dataset population based on its features. The goal is to determine the least number of features that produce the least amount of error in classifying samples and forecasting CVDs patients. Heightening the accuracy of the input data for the ML method should enable the learning model to recognize precise patterns for the diagnosis and prognosis of CVDs.
- Future researchers ought to employ methods like Ant Colony Optimization Algorithms and Particle Swarm Optimization to enhance model efficacy. To attain superior forecast accuracy, they should adopt hybrid and ensemble models, and explore novel research opportunities in this domain through the application of predictive data mining techniques in medical diagnosis.

## Data Availability

Not applicable.

## Abbreviations

CVD: Cardiovascular disease
COVID-19: Coronavirus disease of 2019
MERS: Middle East respiratory syndrome
KNN: K-nearest neighbors
SVM: Support vector machine
RF: Random forest
ANN: Artificial neural network
LR: Logistic regression
SGD: Stochastic gradient descent
DBSCAN: Density-based spatial clustering of applications with noise
CI: Cardiac insufficiency
HF: Heart failure
CKD: Chronic kidney disease
SDoH: Social determinants of health
ML: Machine learning
HIT: Health information technology
EHR: Electronic health records
GBC: Gradient boosting classifier
DT: Decision tree
SFS: Selected feature selection
ETC: Extra tree classifier
SMOTE: Synthetic minority oversampling technique
FCMIM: Fuzzy c-means index matrix
HR: Heart rate
AI: Artificial intelligence
ECG: Electrocardiography
LDL: Low density lipoprotein
HDL: High density lipoprotein
C: Cluster
